# Inequalities in unmet health care needs under universal health insurance coverage in China

**DOI:** 10.1186/s13561-023-00473-4

**Published:** 2024-01-02

**Authors:** Jingxian Wu, Yongmei Yang, Ting Sun, Sucen He

**Affiliations:** 1 School of Economics and Finance, Xi’an Jiaotong University, Xi’an, Shaanxi P.R. China; 2 School of Health and Management, Wenzhou Medical University, Wenzhou, Zhejiang P.R. China

**Keywords:** Social health insurance, Unmet health care needs, Inequality, Concentration index, Decomposition, Financial and non-financial constraints

## Abstract

**Background:**

Expanding health insurance is a critical step towards universal health coverage due to its positive effect on reducing unmet health care needs and enhancing equitable access to health care. Despite previous studies on the socioeconomic factors associated with unmet health care needs, few studies have analysed the inequalities in such needs and the impact of universal health insurance coverage on addressing them. This study aimed to measure the contribution of social health insurance (SHI) coverage to inequalities in financially and non-financially constrained unmet health care needs among middle-aged and elderly Chinese adults.

**Methods:**

The study data were obtained from the China Health and Retirement Longitudinal Study (2011–2015). A total of 11,592 respondents reporting outpatient care needs and 6320 reporting inpatient care needs were included. The concentration index (CI) was employed to measure the extent of income-related inequalities in unmet health care needs. A decomposition method based on a probit model was used to investigate the contribution of SHI to the inequalities.

**Results:**

The incidence rates of unmet outpatient needs due to financial and non-financial constraints were 4.68% and 24.78%, respectively; these rates were 18.69% and 15.73% for unmet inpatient needs. The CIs of unmet outpatient needs due to financial and non-financial constraints were − 0.1872 and 0.0195, respectively; these values were − 0.1558 and 0.0352 for unmet inpatient needs. The percentages of the contribution of SHI to the CIs of financially constrained unmet outpatient and inpatient needs were 0.2639% and 1.8898%, respectively. Moreover, the percentages of the contribution of SHI to the CIs of non-financially constrained unmet outpatient and inpatient needs were − 0.4513% and − 6.4192%, respectively.

**Conclusion:**

The universal coverage of SHI in China increased pro-poor inequalities in financially constrained unmet health care needs but decreased pro-rich inequalities in non-financially constrained unmet needs. Additionally, the contribution of SHI to inequalities in financially constrained unmet needs for inpatient care was stronger than that for outpatient care. Policy-makers are advised to introduce favourable reimbursement policies for patients with poor socioeconomic conditions and address both financial and non-financial barriers to promote equitable access to health care for the entire population.

## Introduction

Equal access to health care means that individuals with equal needs have equal access opportunities regardless of their social and economic conditions [1]. Achieving universal health coverage (UHC) in many low- and middle-income countries remains challenging [2]. Both financial and non-financial obstacles can hinder people from accessing health care services when ill, resulting in deteriorated health and diminished quality of life [3–5]. Many developing countries have recently introduced social health insurance (SHI) schemes to enhance equal access to basic health care for their populations [6]. SHI is a public health care financing mechanism committed to ensuring all citizens have equitable access to affordable basic health care by reducing barriers to health care for low-income individuals [7]. In this regard, one of the objectives of extending SHI coverage is to decrease inequalities in health care utilization due to financial difficulties, consequently safeguarding the population’s health and advancing the realization of UHC.

With the implementation of the New Medical Reform (NMR) in 2009, the Chinese government established an SHI system with a political commitment to “*provide all its citizens access to equitable and quality basic health care by 2020*” [8]. The SHI system comprises an employment-based SHI scheme for formally employed or retired residents (i.e., the Urban Employee Basic Medical Insurance) and two nonemployment-based SHI schemes for rural and urban residents without formal employment (i.e., the New Rural Cooperative Medical Scheme for rural residents and the Urban Resident Basic Medical Insurance for urban residents). A decade after the NMR, China has effectively accomplished universal health insurance coverage for its population, with over 95% of individuals covered by the SHI schemes. Government subsidies have consistently grown, gradually expanding insurance benefits for policyholders [9].

Ample evidence shows that China’s SHI schemes effectively enhance the timely use of health care services by minimizing out-of-pocket (OOP) payments, particularly for individuals with lower socioeconomic status [10, 11]. Nevertheless, access to health care in China remains highly unequal. In addition to the disparities caused by the fragmentation of the SHI schemes, which require integration into a unified system [12], inequalities in access to health care were primarily reflected in a pro-rich distribution in service utilization and insurance benefits [13, 14]. Many studies have suggested that patients from high-income households or urban regions, who enjoy better socioeconomic conditions, tend to utilize more health care services and receive greater benefits from SHI reimbursement than their less advantaged counterparts [14–16]. Furthermore, because of the disparities in SHI compensation policies between outpatient and inpatient services, unequal access among individuals with different socioeconomic backgrounds is more pronounced in inpatients than outpatients [7]. In general, the impact of the SHI schemes on promoting equal access to health care has been unsatisfactory, failing to meet expectations regarding the reduction of inequalities in the use of health care services.

As the demand for health care services rises due to population ageing and advancements in medical technology, the effectiveness of China’s SHI funding, which operates on low financing levels, is being increasingly scrutinized [17]. Despite the large percentage of the population covered by SHI schemes, unmet health care needs remain a critical challenge in ensuring equal access to health care. This issue needs to be urgently addressed; failure to do so will result in an unhealthy population and undermine health equity, impeding progress towards UHC [18]. Unmet health care needs refer to the subjective perception of not receiving appropriate medical services when necessary [19]. This indicator has been widely adopted as a measure of equal access to health care [20]. Assessing the degree of underuse of health care offers a more accurate reflection of the accessibility of health care than investigating the actual use of health care services because it can genuinely and concretely represent a patient’s overall health care needs [21, 22]. Identifying the extent of unmet needs can provide valuable insights into the degree to which patients are not receiving necessary care and the factors contributing to the gap between health care needs and provision [23, 24]. This information can be used to develop targeted and effective interventions to improve access to health care services. Even in the context of universal health insurance coverage, unmet needs persist when financial or non-financial barriers prevent individuals from seeking necessary health care [25, 26].

Typically, unmet health care needs arise due to the limited availability of health care services, such as long waiting times, distance to providers, and affordability issues, such as the cost of care at the time of need [4]. Numerous studies have examined the prevalence of unmet health care needs by analysing self-reported instances of foregone or postponed needed health care [5, 27, 28]. Factors associated with the nonuse of health care services have also been investigated, including age, sex, education level, employment status, health status, the inadequate supply of high-quality service, high OOP medical payments, a lack of financial support (such as health insurance or Medicaid), and transportation inconvenience [18, 24, 29–32]. Notably, a lack of health insurance and low socioeconomic conditions are strongly associated with a higher likelihood of experiencing unmet health care needs [31, 33]. Unmet needs for health care services are most pronounced in the most socioeconomically disadvantaged individuals, such as those from low-income households or rural regions [23, 24].

It has been theoretically established that disparities in these associated factors with healthcare service utilization will cause inequalities in unmet health care needs across different socioeconomic groups [33]. However, empirical evidence is lacking. Previous studies have mostly focused on comparing the prevalence of the nonuse of health care services across different socioeconomic groups [18, 33–36]. Few studies have quantified the extent of inequalities in unmet health care needs and elucidated the role of health insurance and other socioeconomic factors in these disparities by calculating and decomposing inequality indices. For instance, Kim et al. [37] calculated the slope and relative indices of inequality and found little evidence of the impact of the expansion of health insurance to include adult dental care coverage on reducing income-based oral health inequalities in Korea. However, importantly, their research was limited to a specific area, namely, dental care, and may not represent the broader impact of health insurance expansion on socioeconomic inequalities in health care access. Coube et al. [23] provided insights into the contribution of private health insurance (PHI) to inequalities in unmet needs for health care services and medication through their decomposition of the concentration index (CI). However, their study’s generalizability is limited due to PHI’s restricted coverage of the Brazilian population. Moreover, most previous studies on unmet health care needs have failed to differentiate between financially and non-financially constrained unmet needs, leading to overly broad analyses [31, 36–38].

In summary, despite the provision of universal health insurance coverage in China, substantial disparities in health care access remain among different socioeconomic groups. Since income gap is already a serious social problem in China [39], it is crucial to thoroughly examine the income-related inequalities in unmet health care needs, placing particular emphasis on the impact of health insurance expansion. This study aims to fill the research gap by examining whether China’s universal coverage of SHI has reduced income-based inequalities in unmet health care needs. We analysed the unmet needs for outpatient and inpatient care separately in accordance with the differentiated SHI reimbursement policies between outpatients and inpatients in China. In contrast to earlier studies that grouped unmet needs due to financial and non-financial reasons together or only analysed those stemming from financial obstacles, we contributed to the previous literature by categorizing unmet needs into two distinct types: those resulting from financial constraints and those arising from non-financial constraints. This allows us to present more precise evidence to expose the inequalities in unmet health care needs and guide the future reform of China’s SHI system. This study’s findings also have significant policy implications for other developing countries facing similar challenges when developing UHC.

The present study used nationally representative survey data to measure income-related inequalities in unmet health care needs and evaluate the role of China’s SHI system in addressing these inequalities. The Introduction section provided background information and reviewed previous studies on this topic. The Methods section describes the research sample, data sources, variable measurements, and analytical methods. We compared the incidence of financially and non-financially constrained unmet needs for outpatient and inpatient care, calculated the CIs to measure income-related inequalities, and used a decomposition method to estimate the contributions of SHI and other socioeconomic factors to the aforementioned inequalities. The Results section presents descriptive statistics of the sample, estimated CIs, and decomposition results. The Discussion section provides an interpretation of the findings and acknowledges the limitations of the present study. Finally, based on the empirical findings, the Conclusion and Policy Implication section discusses the implications for future policy.

## Methods

### Sample selection

Middle-aged and elderly Chinese adults were selected as the analytical samples for this study since previous research has demonstrated that this population is more sensitive to health care costs than younger individuals [40]. Most, if not all, of these individuals experienced economic challenges early in life, making them more frugal and sensitive to price changes, including those related to health care services [41]. This generation has experienced the Cultural Revolution (1966–1976) and the early stages of the Reform and Opening-up Era (late 1970s and early 1980s) and have typically saved a significant portion of their earnings for their children and parents as a result. However, sharp increases in health care costs exacerbate their economic burden, causing them to seek medical attention only when their illnesses become critical [42]. Therefore, SHI reimbursement may alleviate their concerns and encourage them to use health care services promptly. As a result, unmet health care needs in the presence of SHI among middle-aged and elderly individuals warrant special attention.

### Data source

The study data were obtained from the 2011, 2013, and 2015 waves of the China Health and Retirement Longitudinal Study (CHARLS). The CHARLS is a survey of a nationally representative sample of individuals aged 45 and above in China, conducted biennially by the National School of Development at Peking University. The CHARLS provides a wealth of information on socioeconomic status and health conditions and has been widely used in the field of health economics [42]. The national baseline survey of the CHARLS was conducted between June 2011 and March 2012. Samples were selected through multistage probability sampling and covered 28 provinces, 150 counties or districts, and 450 villages or urban communities in China. Ultimately, 17,708 individuals from 10,026 households were interviewed on a range of social, economic, and health circumstances. Three follow-up waves of the survey were conducted in 2013, 2015, and 2018 to track the changes in the same respondents’ circumstances over the preceding two-year period while also incorporating a small share of new respondents, resulting in a total of 18,605, 21,095, and 19,816 individuals over the three waves, respectively. The original CHARLS was approved by the Ethical Review Committee of Peking University, and all the participants signed informed consent documentation at the time of participation [42].

Data from the first three waves (2011, 2013, and 2015) were used in this study as the latest survey conducted in 2018 unfortunately did not contain information on unmet health care needs. The integrity of the raw data was checked, and respondents with too many missing variables were excluded. Respondents reporting a need for outpatient care (those who became ill) in the previous month or inpatient care (those who had inpatient care recommended by a doctor) in the previous year were selected as research samples. In total, 11,592 respondents reporting a need for outpatient care and 6320 reporting a need for inpatient care were included.

### Measurement of outcome variables

The outcome variables in this study measured whether respondents had experienced unmet health care needs. Due to the disparities in SHI compensation policies between outpatient and inpatient services in China [7], we analysed unmet needs for outpatient and inpatient care separately. Each was further classified into two types based on the primary reasons [26].

Unmet needs for outpatient care due to financial and non-financial constraints:

Respondents were identified as having unmet needs for outpatient care if they reported being ill in the previous month but did not visit any health care institutions. This is a recoded variable derived from two questions in the CHARLS survey: “*(1) Have you been ill in the last month? (2) In the last month, have you visited a public hospital, private hospital, public health centre, clinic, health worker’s or doctor’s practice, or been visited by a health worker or doctor for outpatient care?*” Those who answered “yes” to the first question but “no” to the second were classified as having unmet needs for outpatient care.

These respondents were further classified into two categories based on their primary reasons for not seeking outpatient care (i.e., financial or non-financial). The CHARLS survey identified eight reasons for not seeking outpatient care: (1) already under treatment, (2) illness is not serious and does not merit treatment, (3) no money, (4) no time, (5) inconvenient traffic, (6) poor service in the hospital, (7) treatment is not useful, and (8) others. Respondents who reported a lack of money as the main reason were classified as “having unmet needs for outpatient care due to financial constraints” (coded as 1 for “yes” and 0 for “no”). Those who cited any of the other reasons were classified as “having unmet needs for outpatient care due to non-financial constraints” (coded as 1 for “yes” and 0 for “no”).

Unmet needs for inpatient care due to financial and non-financial constraints:

We identified those for whom inpatient care had been recommended by a doctor in the previous year but had not been hospitalized as having unmet outpatient needs. This information was obtained from the following question in the CHARLS survey: “*In the past year, did a doctor suggest that you needed inpatient care, but you were not hospitalized?*”

Similarly, respondents with unmet needs for inpatient care were further classified into two groups based on their primary reasons for not seeking inpatient care (i.e., financial or non-financial). The reasons for not seeking inpatient care included (1) not enough money, (2) not willing to go to the hospital, (3) poor hospital quality leading to a belief that the hospital was unlikely to cure problems, (4) severity of illness leading to a belief that care was unlikely to cure the illness, (5) no ward was available, and (6) others. Respondents who reported a lack of money as the primary reason were classified as “having unmet needs for inpatient care due to financial constraints” (coded as 1 for “yes” and 0 for “no”). Those who cited any of the other reasons were classified as “having unmet needs for inpatient care due to non-financial constraints” (coded as 1 for “yes” and 0 for “no”).

### Measurement of covariate variables

Our empirical analysis was guided by Andersen’s conceptual model of access to health care [43]. According to Andersen’s healthcare service use behavioural model, we used a multifaceted approach to identify the factors influencing the unmet health care needs among Chinese middle-aged and elderly adults [44]. Previous research has identified several covariate variables as potential correlates of unmet health care needs and health care service utilization [24, 29–32, 45, 46]. Predisposing factors are the individual sociodemographic characteristics of people who tend to use health care services before getting ill, including age, sex, education level, job status, and living status. Enabling factors are resources that can indirectly support the adoption of health care services in case of sickness, including household income level, enrolment in SHI, and possession of supplementary PHI. Importantly, the core covariate of the present study was the respondents’ SHI participation. It was measured as whether respondents were insured by any of the SHI schemes in China (coded as 1 for “yes” and 0 for “0”). Need factors are those that can most directly affect health care use. In our study, they included self-perceived needs (self-rated childhood health and self-rated health [SRH]) and actual needs (chronic disease suffering and disability with activities of daily life [ADL]).

Data regarding the geographical accessibility of health care (travel time to the nearest health care institution via the conventional mode of transport), municipal economic level (gross regional product [GRP] per capita of the city where the community sample is located), and community location (urban or rural area, East, Central, or Western China) were also included as covariates in this study. These factors have been shown to affect healthcare-seeking behaviour and are considered potential enabling factors [47]. These factors were chosen due to persistent regional disparities in health care resource allocation, which remain significant obstacles to more equitable access to health care services in China [17]. Currency-related variables (in this case, GRP per capita and annual per capita household income) from 2013 to 2015 were deflated to 2011 levels using the corresponding consumer price index, and a natural logarithm was used to capture the nonlinear relationship between the economic variables and the occurrence of unmet health care needs.

A detailed description of the covariates is shown in Table 1.

**Table 1:** Description of variables

Variables	Description
SHI	The respondent was insured by any of the social health insurance (SHI) schemes; Yes = 1; No^a^ = 0
Age (Years)	Age of the respondent
Gender	Gender of the respondent; Female^a^ = 0; Male = 1
Education level	Highest education level achieved of the respondent; Illiterate^a^ = 0; Primary school = 1; Middle school = 2; High school and above = 3
Marital status	Marital status of the respondent; With a spouse = 1; Without a spouse^a^ (including unmarried, divorced, or widowed) = 0
Living with children	The respondent lived with children; Yes = 1; No^a^ = 0
Job	Job status of the respondent; Without a job (including get retired)^a^ = 0; Agricultural job = 1; Non-agricultural job = 2
Income (RMB)	The annual per capita household income
Ln income	The natural logarithm of the sum of the annual per capita household income and 1
Childhood health	Score of self-rated childhood health status of the respondent (range: 1–5); Much healthier = 1; Somewhat healthier = 2; Average = 3; Somewhat less healthy = 4; Much less healthy = 5
SRH	Score of self-rated health (SRH) status of the respondent (range: 1–5); Very good = 1; Good = 2; Fair = 3; Poor = 4; Very Poor = 5
Chronic diseases	The respondent ever suffered from chronic diseases; Yes = 1; No^a^ = 0
ADL disability	The respondent had disability with activities of daily life (ADL); Yes = 1; No^a^ = 0
PHI	The respondent was insured by supplementary private health insurance (PHI); Yes = 1; No^a^ = 0
Travel time (Minutes)	Travel time to the nearest healthcare institution by conventional transportation
Urban or rural	Urban or rural area according to National Statistical Bureau; Urban = 1; Rural^a^ = 0
City GRP (RMB)	Gross Regional Product (GRP) per capita of the sample city in the previous year
ln city GRP	The natural logarithm of GRP per capita of the sample city in the previous year
Region	Region of the sample community; East China^a^ = 0; Central China = 1; West China = 2

Note: ^a^ Reference group

### Measurement and decomposition method of inequalities in unmet health care needs

According to the sample’s descriptive statistics presented in Table 2, we computed the CI to assess income-related inequalities in unmet health care needs. CI was developed by Wagstaff and Van Doorslaer and is the most commonly used indicator to measure inequality in health care needs and utilization [48]. CI accounts for the sampled respondents’ socioeconomic status (in this case, income level) and is sensitive to changes in household distribution across socioeconomic groups. The concentration curve plots the cumulative percentage of the health care demanders ranked by annual per capita household income from the poorest to the richest (*x-axis*) against the cumulative percentage of the occurrence of unmet health care needs (*y-axis*). The CI is twice the area enclosed by the concentration curve and absolute equality line [49] and ranges from − 1 to + 1; the closer it is to zero, the more equitable the distribution will be. A positive CI implies that unmet health care needs are more concentrated among richer respondents, while a negative CI indicates the opposite. A CI of zero indicates an equal distribution of unmet health care needs across different income groups [49]. Eq. (1) illustrates the CI calculation:

1 $$CI = \frac{2}{\mu }{\mathop{\rm cov}} \left( {{y_i},{r_i}} \right)$$

where ${y}_{i}$ denotes the indicators measuring unmet health care needs, ${r}_{i}$ is the fractional rank of respondents reporting health care needs in terms of the income distribution, and $\mu$ represents the mean occurrence of unmet health care needs.

A decomposition method of CI was employed in this study following the approach proposed by Wagstaff et al. [50] to analyse the contributions of SHI and other covariates to income-related inequality in the occurrence of unmet health care needs. We used a probit regression model to decompose the CI, considering the dichotomous nature of having unmet needs. As the probit model is nonlinear, we estimated the marginal effect evaluated at the covariate means to calculate the linear approximation to the probit model. The specific regression model can be expressed as follows:

2 $${y_i} = \delta + {\sum\nolimits_k}{\gamma _k}{z_k} + {\varepsilon _i}$$

In Eq. (2), ${y}_{i}$ indicates whether the respondent has incurred unmet health care needs, ${z}_{ki}$ represents the covariates, and $\delta$, ${\gamma}_{k}$, and ${\varepsilon}_{i}$ denote the constant term, marginal effect, and disturbance terms, respectively. The method of decomposition of CI can be specified as follows:

3 $$CI = {\sum\nolimits_k}\frac{{{\lambda _k}{{\bar z}_k}}}{\mu }{C_k} + \frac{{G{C_\varepsilon }}}{\mu }$$

where ${\bar {z}}_{k}$ denotes the mean of each covariate, ${C}_{k}$ represents the CI of each covariate, ${\gamma }_{k}{\bar {z}}_{k}/\mu$ is the elasticity of CI, and $G{C}_{\varepsilon }/\mu$ is the error term [34].

All analyses were performed using Stata software, Version 16.1.

## Results

### Descriptive statistics

The descriptive statistics of the covariate variables for respondents reporting needs for outpatient or inpatient care are presented in Table 2.

**Table 2:** Summary statistics of covariate variables

Variables	Respondents reporting needs for outpatient care		Respondents reporting needs for inpatient care	
	Mean (N)	SD (%)	Mean (N)	SD (%)
Sample size	11,592	100%	6320	100%
SHI				
Yes	11,274	97.26%	6185	97.86%
No^a^	318	2.74%	135	2.14%
Age	61.45	0.09	63.13	0.12
Gender				
Male	4938	42.60%	2931	46.38%
Female^a^	6654	57.40%	3389	53.62%
Education level				
Illiterate^a^	3345	28.86%	1817	28.75%
Primary school	4944	42.65%	2693	42.61%
Middle school	2133	18.40%	1168	18.48%
High school and above	1170	10.09%	642	10.16%
Marital status				
With a spouse^a^	9842	84.90%	5306	83.96%
Without a spouse	1750	15.10%	1014	16.04%
Living with children				
Yes	7052	60.84%	3522	55.73%
No^a^	4540	39.16%	2798	44.27%
Job				
Without a job^a^	4137	35.69%	2926	46.30%
Agricultural job	6341	54.70%	2890	45.73%
Non-agricultural job	1194	9.61%	542	7.97%
Income	10086.14	186.82	10046.36	209.06
Ln income	8.65	0.01	8.66	0.01
Childhood health	2.83	0.01	2.76	0.01
SRH	3.65	0.01	3.74	0.01
Chronic diseases				
Yes	6814	58.78%	3891	61.57%
No^a^	4778	41.22%	2429	38.43%
ADL disability				
Yes	2601	22.44%	1866	29.53%
No^a^	8991	77.56%	4454	70.47%
PHI				
Yes	206	1.78%	104	1.65%
No^a^	11,386	98.22%	6216	98.35%
Travel time	25.40	0.18	26.79	0.23
Urban or rural				
Urban	4036	34.82%	2348	37.15%
Rural^a^	7556	65.18%	3972	62.85%
City GRP	36352.24	205.43	36618.77	268.82
ln city GRP	10.35	0.01	10.36	0.01
Region				
East China^a^	3671	31.67%	1992	31.52%
Central China	3294	28.42%	1793	28.37%
West China	4627	39.92%	2535	40.11%

Note: ^a^ Reference group

Among the 11,592 respondents in need of outpatient care and the 6320 respondents in need of inpatient care, over 97% were covered by SHI, and under 2% were covered by PHI. The mean age of respondents reporting outpatient and inpatient needs was 61.45 and 63.13 years, respectively, with a higher percentage of females reporting health care needs. Over 42% of the respondents had a primary education. Approximately 84% of the respondents had a spouse, and over half lived with children. More unemployed respondents reported unmet needs for inpatient care than outpatient care. The annual per capita household income was approximately 10 thousand RMB. The mean SRH score of outpatient need reporters was 3.65, and that of inpatient need reporters was 3.74, indicating a poorer health status in the latter. Over one-fifth of the outpatient need reporters (22.44%) experienced disabilities with their ADL, and the proportion of inpatient need reporters was as high as 29.53%. Over 58% of the outpatient need reporters and 61% of the inpatient need reporters were diagnosed with chronic diseases. The average travel time to the nearest health care institution was under 30 min for both outpatient and inpatient need reporters, indicating the geographical accessibility of health care facilities. Over 60% of the respondents were from rural households, which aligns with China’s demographic distribution, with a greater percentage of rural residents in the total population.

### Incidence of unmet health care needs

The incidence of unmet outpatient and inpatient care needs is presented in Table 3. Among the 11,592 respondents reporting needs for outpatient care, 4.68% (543 respondents) had unmet outpatient needs due to financial reasons, while 24.78% (2873 respondents) had unmet needs due to non-financial reasons. The results showed a significantly higher incidence of unmet outpatient needs due to non-financial barriers than financial barriers, with the former approximately six times greater than the latter. The total incidence of unmet outpatient care needs was approximately 30%. Among the 6,320 respondents who reported needs for inpatient care, 18.69% (1181 respondents) experienced unmet needs due to insufficient money, while 15.73% (994 respondents) experienced unmet needs due to non-financial constraints. The total incidence of unmet inpatient needs (34.42%) was higher than that of unmet outpatient needs.

**Table 3:** Incidence of unmet health care needs

Unmet reasons	Incidence of unmet needs for outpatient care		Incidence of unmet needs for inpatient care	
	N	%	N	%
Unmet due to financial constraints	543	4.68%	1181	18.69%
Unmet due to non-financial constraints	2873	24.78%	994	15.73%

### Inequalities in unmet health care needs

**Fig. 1:**
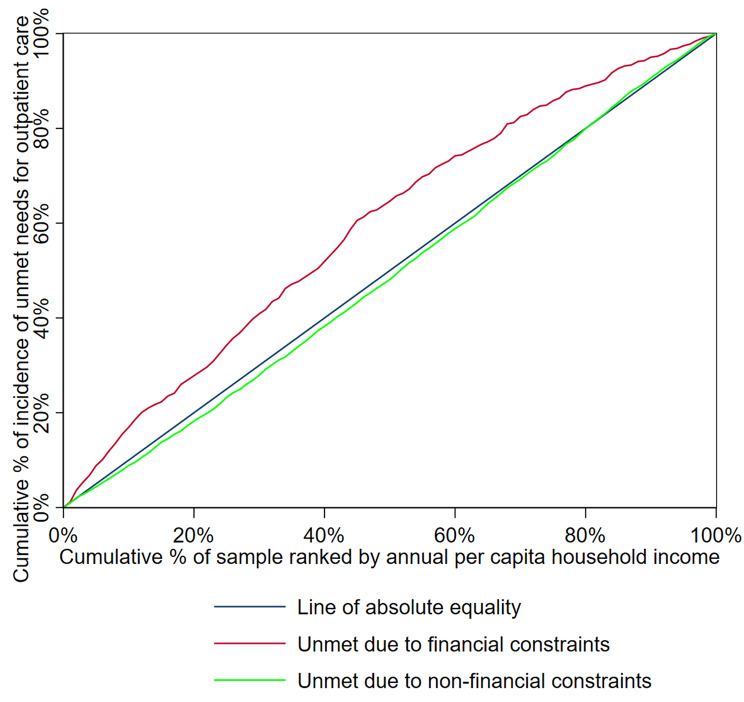
Concentration curve of unmet needs for outpatient care

**Fig. 2:**
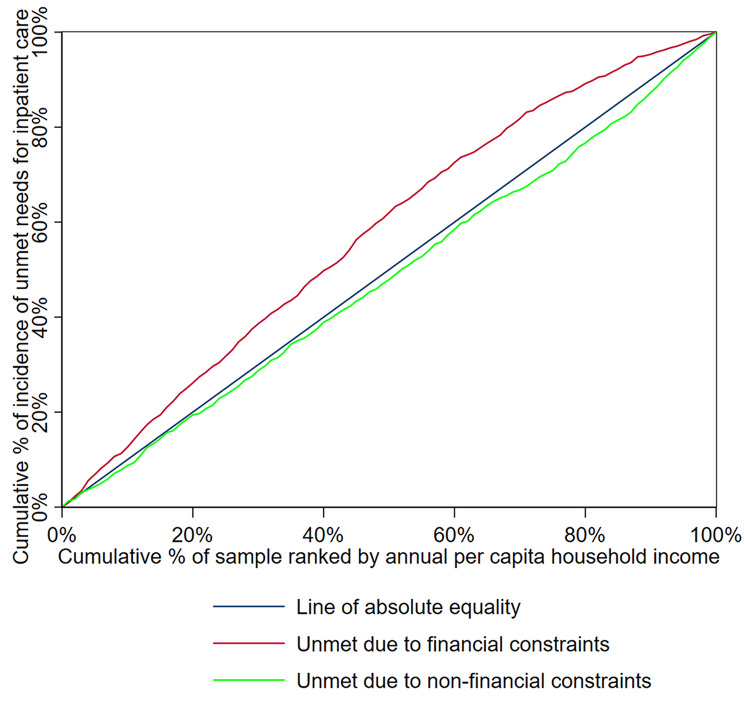
Concentration curve of unmet needs for inpatient care

The CI for unmet needs for outpatient and inpatient care is presented in Table 4, and the concentration curves of the unmet needs for outpatient and inpatient care are shown in while Figs. 1 and 2, respectively.

**Table 4:** Inequalities in unmet health care needs

Unmet reasons	CIs for unmet needs for outpatient care	CIs for unmet needs for inpatient care
Unmet due to financial constraints	-0.1872 (-0.2322, -0.1423)	-0.1558 (-0.1837, -0.1279)
Unmet due to non-financial constraints	0.0195 (0.0014, 0.0376)	0.0352 (0.0016, 0.0687)

Note: 95% confidence interval in the parentheses

The CI for the incidence of unmet needs due to financial constraints for both outpatient and inpatient care was negative (-0.1872 and − 0.1558, respectively), indicating pro-poor inequalities in the distribution of financially constrained unmet needs across different income groups. The concentration curves for both outpatient (Fig. 1) and inpatient care (Fig. 2) were above the line of absolute equality, further demonstrating that these unmet health care needs were concentrated among the most economically disadvantaged individuals. In contrast, the CIs for unmet needs due to non-financial constraints were positive (0.0195 for outpatient care and 0.0352 for inpatient care), indicating pro-rich inequalities in the distribution of non-financially constrained unmet needs. The concentration curves of unmet health care needs due to non-financial reasons fell below the line of absolute equality, as depicted in Figs. 1 and 2.

### Contribution of SHI to inequalities in unmet health care needs

The decomposition results for inequalities in unmet needs for outpatient and inpatient care are presented in Tables 5 and 6, respectively.

**Table 5:** Decomposition of inequalities in unmet needs for outpatient care

Variables	Unmet due to financial constraints				Unmet due to non-financial constraints			
	Marginal effect	Elasticity	Cont.^b^	Per.^c^ (%)	Marginal effect	Elasticity	Cont.^b^	Per.^c^ (%)
SHI, yes	-0.0328***	-0.6817	-0.0005	0.2639	-0.0310	-0.1218	-0.0001	-0.4513
Age	-0.0007**	-0.8873	0.0075	-3.9934	0.0002	0.0418	-0.0004	-1.7988
Gender, male	-0.0028	-0.0253	-0.0005	0.2547	0.0195**	0.0335	0.0006	3.2324
Education								
Illiterate^a^								
Primary school	0.0028	0.0259	-0.0010	0.5611	0.0112	0.0193	-0.0008	-4.0049
Middle school	-0.0098	-0.0384	-0.0053	2.8334	0.0042	0.0031	0.0004	2.1804
High school and above	-0.0176**	-0.0380	-0.0144	7.7173	-0.0064	-0.0026	-0.0010	-5.0395
Marital status, with a spouse	-0.0135**	-0.2445	-0.0026	1.4010	-0.0045	-0.0154	-0.0002	-0.8433
Living with children, yes	-0.0103**	-0.1333	0.0051	-2.7024	0.0078	0.0190	-0.0007	-3.6965
Job								
Without a job^a^								
Agricultural job	0.0104**	0.1212	-0.0117	6.2702	0.0193***	0.0426	-0.0041	-21.0903
Non-agricultural job	-0.0011	-0.0024	-0.0006	0.3432	0.0606***	0.0252	0.0067	34.4356
Ln income	-0.0075***	-1.3900	-0.0975	52.0865	0.0094**	0.3267	0.0229	117.1619
Childhood health	0.0018	0.1068	-0.0018	0.9426	0.0138***	0.1576	-0.0026	-13.3134
SRH	0.0147***	1.1465	-0.0197	10.5151	-0.0075	-0.1111	0.0019	9.7554
Chronic diseases, yes	-0.0001	-0.0015	0.0000	-0.0263	-0.0462***	-0.1096	0.0037	18.6860
ADL disability, yes	0.0271***	0.1296	-0.0164	8.7894	-0.0071	-0.0065	0.0008	4.2017
PHI, yes	-0.0212	-0.0081	-0.0017	0.9105	-0.0126	-0.0009	-0.0002	-0.9794
Travel time	0.0002*	0.1069	-0.0044	2.3568	0.0001	0.0071	-0.0003	-1.4904
Urban or rural, urban	-0.0064	-0.0479	-0.0106	5.6878	0.0343***	0.0482	0.0107	54.7256
Ln city GRP	-0.0007	-0.1535	-0.0013	0.6895	-0.0324***	-1.3527	-0.0114	-58.1411
Region								
East China^a^								
Central China	-0.0002	-0.0012	0.0001	-0.0282	0.0099	0.0114	-0.0005	-2.5605
West China	0.0224***	0.1906	-0.0163	8.6951	0.0635***	0.1022	-0.0087	-44.6354

Note: ^a^ Reference group; ^b^ Contribution to CI; ^c^ Percentage of contribution to CI; * *p* < 0.1; ** *p* < 0.05; *** *p* < 0.01

**Table 6:** Decomposition of inequalities in unmet needs for inpatient care

Variables	Unmet due to financial constraints				Unmet due to non-financial constraints			
	Marginal effect	Elasticity	Cont.^b^	Per.^c^ (%)	Marginal effect	Elasticity	Cont.^b^	Per.^c^ (%)
SHI, yes	-0.1349***	-0.7063	-0.0029	1.8898	-0.0873***	-0.5432	-0.0023	-6.4192
Age	-0.0049***	-1.6692	0.0060	-3.8877	0.0007	0.2737	-0.0010	-2.8151
Gender, male	-0.0197*	-0.0489	-0.0008	0.5192	-0.0172*	-0.0506	-0.0008	-2.3711
Education								
Illiterate^a^								
Primary school	0.0052	0.0118	-0.0006	0.3993	0.0001	0.0003	0.0000	-0.0376
Middle school	-0.0142	-0.0141	-0.0023	1.4646	0.0421**	0.0495	0.0080	22.7932
High school and above	-0.0419**	-0.0228	-0.0087	5.6192	0.0638***	0.0412	0.0158	44.8766
Marital status, with a spouse	-0.0175	-0.0785	-0.0004	0.2670	-0.0105	-0.0560	-0.0003	-0.8417
Living with children, yes	-0.0078	-0.0234	0.0011	-0.6811	-0.0049	-0.0172	0.0008	2.2142
Job								
Without a job^a^								
Agricultural job	0.0643***	0.1574	-0.0169	10.8710	0.0114	0.0331	-0.0036	-10.1128
Non-agricultural job	0.0522***	0.0240	0.0058	-3.7374	0.0202	0.0110	0.0027	7.5982
Ln income	-0.0316***	-1.4659	-0.1034	66.4359	0.0047	0.2585	0.0182	51.7454
Childhood health	0.0071	0.1046	-0.0019	1.2379	-0.0036	-0.0637	0.0012	3.3297
SRH	0.0489***	0.9780	-0.0157	10.0944	0.0041	0.0964	-0.0015	-4.3958
Chronic diseases, yes	0.0242**	0.0797	-0.0024	1.5588	-0.0078	-0.0306	0.0009	2.6452
ADL disability, yes	0.0577***	0.0912	-0.0097	6.2585	-0.0096	-0.0181	0.0019	5.4748
PHI, yes	-0.0682	-0.0060	-0.0013	0.8471	-0.0087	-0.0009	-0.0002	-0.5645
Travel time	0.0008***	0.1141	-0.0051	3.2792	-0.0001	-0.0129	0.0006	1.6346
Urban or rural, urban	-0.0143	-0.0283	-0.0066	4.2244	-0.0177	-0.0418	-0.0097	-27.5319
Ln city GRP	0.0167	0.9281	0.0072	-4.6460	0.0217*	1.4322	0.0112	31.6636
Region								
East China^a^								
Central China	-0.0108	-0.0164	0.0006	-0.3829	0.0010	0.0017	-0.0001	-0.1800
West China	-0.0154	-0.0330	0.0023	-1.4920	0.0346**	0.0882	-0.0062	-17.5875

Note: ^a^ Reference group; ^b^ Contribution to CI; ^c^ Percentage of contribution to CI; * *p* < 0.1; ** *p* < 0.05; *** *p* < 0.01

As shown in Table 5, participation in SHI was significantly associated with a reduction in the likelihood of experiencing unmet needs for outpatient care due to financial constraints (*p* < 0.01). However, the association between SHI participation and unmet outpatient needs due to non-financial constraints was not significant (*p* > 0.1) despite a negative marginal effect. By decomposing the CI, it was found that the percentages of SHI’s contribution to the CI for unmet outpatient needs due to financial and non-financial constraints were 0.2639% and − 0.4513%, respectively. These results indicated that SHI increased pro-poor inequalities in financially constrained unmet needs for outpatient care but decreased pro-rich inequalities in unmet outpatient needs due to non-financial reasons.

Regarding unmet needs for inpatient care, as shown in Table 6, SHI participation was significantly associated with a decrease in the incidence of having unmet inpatient needs due to both financial and non-financial constraints (*p* < 0.01). The percentages of SHI’s contribution to the CI due to financial and non-financial constraints were 1.8898% and − 6.4192%, respectively. The results showed that SHI increased pro-poor inequalities in unmet inpatient needs due to financial barriers but reduced pro-rich inequalities in unmet inpatient needs due to non-financial reasons, similar to the contribution of SHI to the inequalities in unmet needs for outpatient care.

**Fig. 3:**
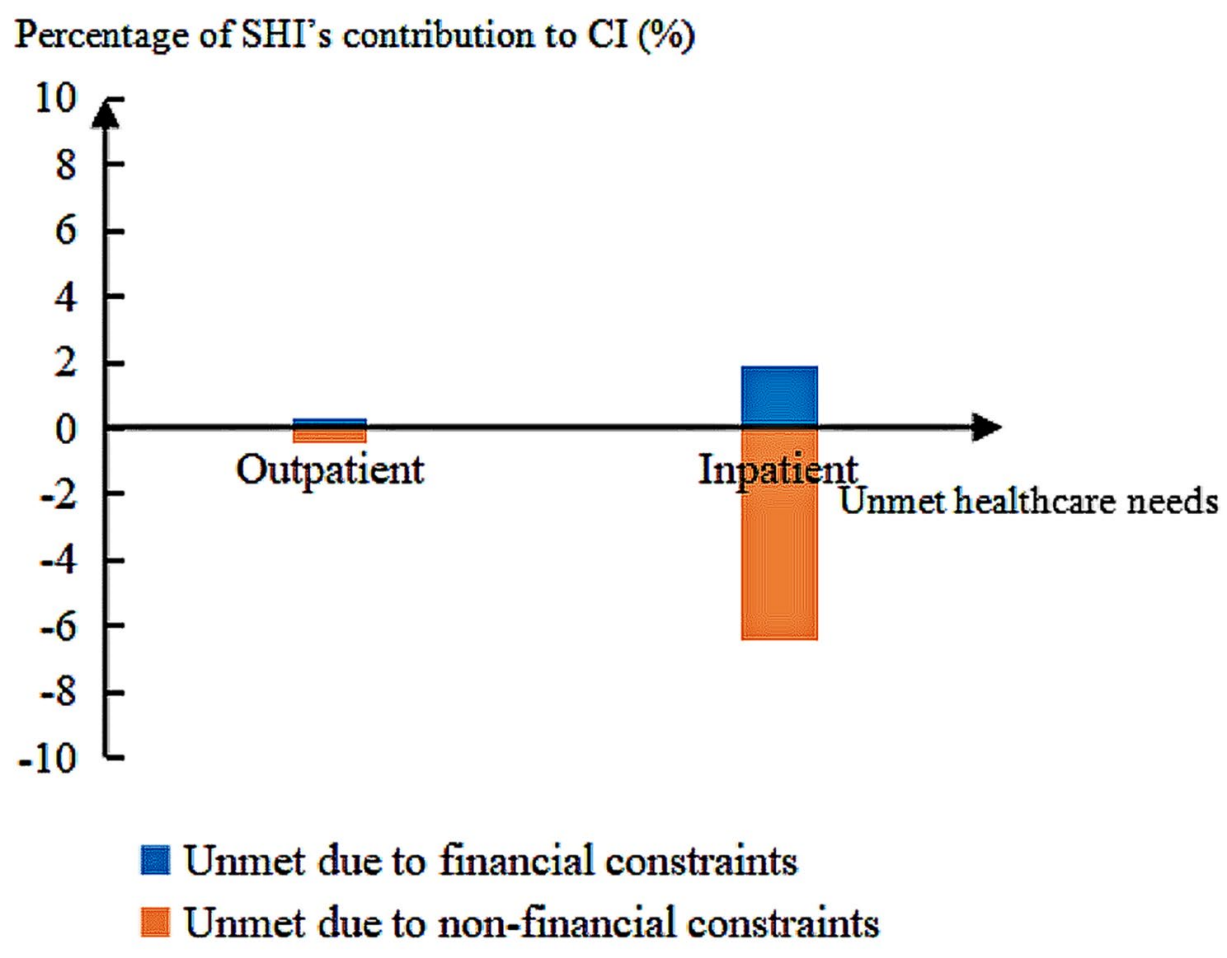
Percentage of SHI’s contribution to inequalities in unmet health care needs

When comparing SHI’s contribution to the inequalities in unmet outpatient care with that in unmet inpatient care (Fig. 3), we found that the percentage of SHI’s contribution to inequality in unmet inpatient care needs was more significant than that of unmet outpatient care needs, regardless of the reason for the unmet need.

### Contribution of other covariates to inequalities in unmet health care needs

The results of the analyses of other factors affecting unmet outpatient needs are also shown in Table 5. The findings indicate that the contribution of these factors varies depending on the reason for the unmet outpatient needs. Specifically, household income level was negatively associated with unmet outpatient needs due to financial constraints (*p* < 0.01) but positively associated with unmet outpatient needs for non-financial reasons (*p* < 0.05). Formal employment in non-agricultural jobs was positively associated with unmet outpatient needs due to non-financial reasons (*p* < 0.01). Poor SRH status and ADL disability were significantly associated with an increase in unmet outpatient needs due to financial constraints (*p* < 0.01), while having a chronic disease was significantly associated with a decrease in unmet outpatient needs for non-financial reasons (*p* < 0.01). Additionally, respondents residing in economically disadvantaged regions, such as rural areas, areas with lower municipal levels, or Western China, reported a higher probability of having unmet outpatient needs for non-financial reasons than their counterparts living in richer regions, such as urban areas, higher municipal economic level, or East or Central China. Regarding the contribution of these factors to the inequalities in unmet outpatient needs, most of the inequality in incurring unmet needs for outpatient care due to financial constraints was attributed to household income level (52.09%), SRH score (10.51%), and education level (10.54%), whereas most of the inequality in incurring unmet needs for outpatient care due to non-financial constraints was related to household income level (117.16%), municipal economic level (-58.14%), urban‒rural disparity (54.73%), and regional disparity (-44.64%).

The results of the analyses of other factors related to unmet inpatient needs are presented in Table 6. Household income level was negatively associated with unmet inpatient needs due to financial reasons (*p* < 0.01) but was not significantly related to unmet inpatient needs due to non-financial reasons (*p* > 0.1). Being employed was positively associated with unmet inpatient needs due to financial reasons (*p* < 0.01). Worse SRH status and ADL disability were significantly associated with increased unmet inpatient needs due to insufficient funds (*p* < 0.01). A lack of geographical access to healthcare facilities, as reflected by a longer travel time to the nearest healthcare institution, was related to an increase in unmet needs for inpatient care, not only in totality (*p* < 0.05) but also due to financial constraints (*p* < 0.01). By decomposing the CI, it was found that most of the inequality in incurring unmet needs for inpatient care due to financial constraints resulted from household income level (66.44%) and SRH score (10.09%), whereas most the inequality in incurring unmet needs for inpatient care due to non-financial constraints was attributed to education level (67.56%), household income level (51.75%), municipal economic level (31.66%), and urban‒rural disparity (-27.53%).

## Discussion

The expansion of SHI coverage aims to promote equal access to health care and reduce unmet health care needs. Research has been conducted on the socioeconomic factors associated with unmet health care needs; however, few studies have analysed the inequalities in such needs and the impact of universal health insurance coverage in addressing them. The present study took middle-aged and elderly Chinese adults as the analytical samples. Using a nationally representative survey dataset, we measured the inequalities in unmet needs for outpatient and inpatient care and the contribution of China’s SHI towards addressing them. Unlike previous studies that combined financially and non-financially related unmet needs or only examined those caused by financial barriers, this study classified the unmet needs into two types based on the primary reasons for not seeking medical treatment when ill: unmet needs due to financial constraints and unmet needs due to non-financial constraints. To the best of our knowledge, this is the first study to investigate the relationship between SHI and inequalities in unmet health care needs resulting from different reasons. The study yielded the following findings:

First, the percentage of respondents who reported unmet outpatient needs due to financial reasons (4.68%) was significantly lower than that of those who reported unmet needs due to non-financial barriers (24.78%). However, the percentage of respondents reporting unmet inpatient needs for financial reasons (18.69%) was higher than that of those who reported non-financial constraints (15.73%). The incidence of unmet health care needs was slightly higher in our study than reported in a recent publication from China on individuals aged over 13 [18]. This is likely because our study focused specifically on middle-aged and elderly adults, who are more economically vulnerable and have greater health care needs than young people or people of all ages. Furthermore, our findings indicated a greater prevalence of unmet needs due to financial constraints for inpatient care than outpatient care, consistent with prior research [18]. This outcome can be attributed to the discrepancies in financial burden for health care services between outpatients and inpatients. Compared to outpatient care, inpatient care entails higher overall medical costs and greater OOP expenses despite compensation from SHI. According to data published by the Chinese government, the mean cost per outpatient visit nationwide was approximately 234 RMB in 2015, whereas the average cost per inpatient admission reached 8268 RMB [51]. Although inpatients benefited from a considerably higher co-payment rate than outpatients (approximately 55% for inpatients versus nearly zero for outpatients in 2015 [52]), the expense paid OOP by inpatients remained substantially higher than those for outpatient care. Hence, it is unsurprising that individuals with inpatient needs were more susceptible to experiencing unmet needs resulting from financial barriers than those with outpatient needs.

Regarding outpatient care, non-financial obstacles, such as a lack of time, inconvenient traffic, and self-treatment, were the primary reasons for unmet outpatient needs. Our findings indicated that the involvement of SHI did not significantly decrease the incidence of non-financially constrained unmet outpatient needs (*p* > 0.1). There is an underlying logic behind these non-financial reasons that discourage individuals from seeking medical care in the concept of opportunity cost, particularly the time cost of visiting a doctor. Long waiting times for outpatient treatment [53] and the resultant economic loss indirectly incurred through medical treatment, such as wage loss and travel expenses, are major reasons people choose not to seek outpatient care when sick. This problem is expected to be even more severe for employed individuals than for unemployed or retired individuals [54]. This is because the former usually face a higher opportunity cost of seeing a doctor, given that almost no employers offer leave pay for sickness in China [54]. According to a previous study in Korea [32], economically active individuals were less likely to receive health care services due to “*time* constraints” (the lack of time) caused by heavy workloads in competitive job markets. Our study on Chinese people supports this finding. As shown in Table 5, having either an agricultural or non-agricultural job is significantly associated with an increased likelihood of experiencing unmet outpatient needs due to non-financial barriers. When the illness is not severe, employed patients may opt for self-treatment rather than visiting a doctor [55]. Therefore, Chinese policy-makers should furtherly address these non-financial barriers to alleviate unmet outpatient needs.

Second, this study revealed a pro-poor distribution of unmet health care needs due to financial constraints and a pro-rich distribution of unmet needs due to non-financial barriers. The severity of the inequalities was greater for the pro-poor distribution, as indicated by the absolute values of CI for unmet health care needs due to financial constraints were higher than 0.15, whereas those for unmet needs resulting from non-financial constraints were less than 0.04. The noteworthy pro-poor distribution of financially constrained unmet needs primarily stems from the inability of economically disadvantaged patients to afford health care services. This finding highlights that low-income individuals are more likely to experience unmet health care needs due to cost, consistent with earlier research [24, 37]. In addition to low household income, high OOP payments for health care, which are linked to limited SHI benefits in China, impede economically vulnerable populations from accessing timely healthcare services. The World Health Organization (WHO) recommends a co-payment rate of 70–80% as a reasonable level to address financial barriers and achieve UHC [56]. However, data from the 2015 CHARLS indicate that the actual co-payment rate for Chinese inpatients was only 40–60%, while for outpatients, it was even less than 10% [52]. The limited benefits and high OOP costs resulting from low co-payment rates, high deductibles, and restricted service coverage offered by SHI schemes have hindered low-income people from consuming timely medical services [14]. In contrast, as noted in previous research, those with higher income levels may prioritize other medical treatment-related issues, such as opportunity cost, service quality, and exorbitant medical fees [57]. Consequently, those with lower economic status were more likely to experience unmet health care needs due to financial constraints, while those with higher economic status were more likely to face non-financial constraints. Therefore, policy-makers are advised to tackle both financial and non-financial barriers to ensure equitable access to health care.

Third, we discovered that SHI amplified inequalities in financially constrained unmet health care needs upon decomposing the CI. In line with prior research [18], our study provides evidence that enrolling in SHI is associated with a notable reduction in the likelihood of experiencing financially related unmet needs for outpatient and inpatient care. However, our results further revealed increased inequalities in these unmet needs resulting from SHI participation. This finding is consistent with a previous study on PHI in Brazil [23]. The increase can be attributed to disparities in service utilization and health insurance benefits among patients from various income levels. Earlier research has demonstrated that patients with higher income levels tend to use more health care services and gain greater advantages from the expansion of health insurance expansion than their lower-income counterparts [58–61]. The pro-rich distribution of insurance benefits is observed not only in developing nations such as China [58, 59] and India [60] but also in developed countries such as Canada [61]. As shown in Tables 4 and 5, household income level was significantly associated with having unmet outpatient and inpatient care needs. Thus, the decision to seek health care services following an illness is heavily influenced by one’s income level. Additionally, as previously discussed, the low co-payment rates of China’s SHI schemes hindered financially disadvantaged individuals from assessing necessary health care services and obtaining reimbursements through the program. This problem was especially acute for individuals who required inpatient care, as evidenced by the fact that 34% of patients in our sample chose to disregard their physician’s advice on hospitalization, with over half doing so because they could not afford the cost of hospitalization. Prior research also supports this finding since individuals who are more economically disadvantaged are less likely to be hospitalized, and even if they are, tend to spend less on inpatient treatment than those who are richer [7]. Furthermore, China’s current reimbursement mechanism for SHI schemes is based almost solely on total health care expenses, without considering patients’ ability to pay [62]. Therefore, it is unsurprising that economically disadvantaged enrolees benefit less than those who are richer under equal compensation arrangements between the rich and poor [7, 62]. As a result, instead of reducing the inequalities in unmet health care needs due to financial constraints, SHI schemes have worsened the gaps. Policy makers need to pay special attention to this matter to guarantee that patients with low socioeconomic status have fair and equal access to healthcare services.

In contrast, the expansion of SHI reduced the inequalities in unmet health care needs arising from non-financial barriers, particularly for inpatient care (with a contribution percentage of -6.42%). The expansion of SHI schemes and the healthcare delivery system’s reform measures have helped reduce the disparities in non-financially caused unmet inpatient needs. The reasons individuals chose not to use health services in such cases were primarily related to the quality and quantity of health care services, such as inadequate service quality and a shortage of vacant beds. These supply-side factors are closely linked to the development of healthcare service delivery systems. China’s healthcare delivery transformation comprises two primary components: a primary healthcare-based integrated delivery system and public hospital reform. Since the implementation of NMR in 2009, primary healthcare institutions have experienced significant development through targeted measures [11, 17]. For instance, the Chinese government has made considerable financial investments, increasing the number of beds in primary healthcare institutions from 1320 thousand in 2009 to 1710 thousand in 2019, while the number of healthcare professionals has risen from 1009 thousand to 1615 thousand over the same period [63, 64]. Supportive policies such as the *zero-markup policy* (i.e., no-profit drug policy) in primary healthcare institutions and the National Essential Medicine Policy have been implemented to promote equitable access to basic health care for all citizens [17, 65]. Reforms have also been undertaken in public hospitals, including the removal of drug price markups, fee schedule adjustments, and provider payment and governance restructuring [66]. These measures have improved access to health care services from public hospitals and primary healthcare institutions [66], which explains why the expansion of SHI has been associated with a decrease in the inequality in unmet health care needs caused by non-financial barriers.

Furthermore, this study revealed a greater increase in the inequalities in financially-based unmet needs for inpatient care than outpatient care following enrolment in the SHI schemes. This is likely because China’s SHI system serves as a significant financial safety net for inpatients rather than outpatients [7], largely due to how SHI reimbursements are distributed across the country. The current SHI schemes prioritize reimbursing economic losses incurred during inpatient care for major illnesses while offering less compensation for economic loss due to minor illnesses in outpatient cases [67]. In addition to a higher co-payment rate for inpatients than outpatients, the SHI service packages for outpatients are restricted, as the cost for certain health care services or medications can only be reimbursed in inpatient cases. Previous research has also shown that the SHI schemes in China were ineffective in reducing OOP payments for outpatient care [68]. As a result, on average, only approximately half of SHI-insured outpatients received reimbursement for their most recent outpatient visit, while almost all inpatients received SHI benefits [69]. Consequently, SHI’s contribution to disparities in unmet health care needs due to financial barriers is more significant for outpatients than inpatients. Therefore, the upcoming SHI reform is advised to include an expansion of the service package and an increase in the reimbursement rate for outpatient care.

Our research findings also reveal that disparities in individual health status and household income level are the primary factors contributing to inequalities in unmet health care needs. In line with previous studies [23, 24, 70], our results demonstrate that poorer health status and lower income levels increase the likelihood of unmet health care needs due to financial constraints. For individuals with poor health and economic disadvantages, policies should focus on alleviating the financial burden of medical care to prevent a “*medical poverty trap*” [71] and worsening health outcomes. Our findings also highlight the critical role of regional economic levels in contributing to disparities in unmet health care needs. As in other developing countries [23], persistent regional disparities in China continue to pose significant obstacles to achieving equitable access to health care services due to variations in the distribution of infrastructure, human resources, access to medication, and coverage by national healthcare programs. Research has shown that high-quality medical resources are often allocated to urban and economically developed regions [47]. Hence, narrowing the regional gap in health resource allocation can effectively alleviate unmet health care needs, particularly in outpatient care [18], and the associated inequalities.

This study has several limitations. First, the data on unmet health care needs were gathered through self-report surveys. The unavailability of health record data may lead to an underestimation of the incidence of unmet health care needs. Additionally, we only considered income-based inequalities in unmet health care needs, which may be biased since income is context-specific and purchasing power varies across regions. Furthermore, we only measured SHI status in terms of participation or nonparticipation. Further research is needed to investigate the effect of specific SHI policies on reducing unmet health care needs and improving equitable access to health care for targeted populations. Finally, once the latest follow-up data becomes available, a trend analysis on changes in inequalities in unmet health care needs is necessary.

## Conclusion and policy implications

The present study used a nationally representative survey dataset to examine income-related inequalities in unmet health care needs among middle-aged and elderly Chinese adults and the role of universal SHI coverage in contributing to these inequalities. Our findings indicated that non-financial barriers were the primary obstacle to accessing outpatient services, while both financial and non-financial barriers hindered patients from obtaining doctor-recommended hospitalization. SHI increased pro-poor inequalities in unmet needs due to financial constraints while reducing pro-rich inequalities in unmet health care needs due to non-financial constraints. The impact of SHI on inequalities related to financially constrained unmet needs for inpatient care is more significant than its impact on outpatient care. We concluded that the reduction of income-related inequalities through the universal health insurance coverage provided by China’s SHI system is limited.

Our research contributes to the literature and policy-making process in the following perspectives. First, compared to previous studies, our research considers more comprehensive measures of unmet health care needs. Rather than measuring overall unmet health care needs [31, 36–38], we focused on outpatient and inpatient needs and classified them into two groups based on their primary reasons for being unmet (i.e., financial or non-financial), resulting in a more accurate estimation of unmet needs for health care. Second, by calculating and decomposing the CIs, we found that the universal coverage of China’s SHI system has exacerbated pro-poor inequalities in financially constrained unmet health care needs but has reduced pro-rich inequalities in non-financially constrained unmet needs. Unlike PHI which has limited coverage, publicly-funded health insurance programs’ expansion, such as SHI or public health insurance, can be an effective means to enhance equitable access to health care and improve population health [72]. Unlike prior studies that only compared the prevalence of the non-use of health care services across different socioeconomic groups [18, 33–36], we quantified the extent of inequalities in unmet health care needs and illuminated the role of health insurance and other socioeconomic factors in these disparities. Lastly, we factored in both outpatient and inpatient care’s unmet needs, which involves varying compensation policies under China’s SHI system. We revealed a greater increase in inequalities in financially based unmet needs for inpatient care compared to outpatient care following the enrolment in the SHI schemes. This allows us to offer more precise evidence to guide the reform of China’s SHI system in the future.

The following policy suggestions are proposed based on research findings. First, policy interventions should be implemented to extend SHI compensation coverage for outpatient care and introduce favourable reimbursement policies for inpatients with poor socioeconomic conditions, thus decreasing financial barriers to accessing health care services. Second, addressing non-financial barriers is also crucial, as the high prevalence of non-financial barriers can severely hinder individuals’ access to necessary health care [26]. Policy-makers in China are recommended to implement measures to reduce the time cost of seeking medical treatment, such as building a “*15-Minute City*” and providing after-hours health care services and telehealth services. Lastly, considering the unbalanced regional development in China, it is advisable to increase the number of health care resources and enhance the quality of health care services in socioeconomically disadvantaged areas to ensure equitable access to health care for the entire population.

## Data Availability

The data source of this study was a publicly available database, the China Health and Retirement Longitudinal Study, which was hosted by the National Development Center of Peking University. The data are available at http://charls.pku.edu.cn/index.htm.

## List of abbreviations

ADL: activities of daily life
CHARLS: China Health and Retirement Longitudinal Study
CI: concentration index
GRP: Gross Regional Product
NMR: New Medical Reform
OOP: out-of-pocket
PHI: private health insurance
SD: standard deviation
SHI: social health insurance
SRH: self-rated health
UHC: universal health coverage
