# Correction: Inequalities in unmet health care needs under universal health insurance coverage in China

**DOI:** 10.1186/s13561-024-00494-7

**Published:** 2024-03-09

**Authors:** Jingxian Wu, Yongmei Yang, Ting Sun, Sucen He

**Affiliations:** 1 School of Economics and Finance, Xi’an Jiaotong University, Xi’an, Shaanxi P.R. China; 2 School of Health and Management, Wenzhou Medical University, Wenzhou, Zhejiang P.R. China

**Correction:** ***Health Econ Rev*** **14, 2 (2024)**

10.1186/s13561-023-00473-4

Following the publication of the original article [1], the authors corrected the National Natural Science Foundation of China grant number to (No. 72104196) instead of (No. 7210040666) in Funding section.

The original article [1] has been updated.
